# Fluoxetine modulates breast cancer metastasis to the brain in a murine model

**DOI:** 10.1186/1471-2407-14-598

**Published:** 2014-08-16

**Authors:** Yuriy Shapovalov, Martha Zettel, Sara C Spielman, Stacy A Amico-Ruvio, Emily A Kelly, Grayson O Sipe, Ian M Dickerson, Ania K Majewska, Edward B Brown

**Affiliations:** Department of Neurobiology and Anatomy, University of Rochester School of Medicine & Dentistry, 601 Elmwood Ave, Box 603, Rochester, NY 14642 USA; Department of Biomedical Engineering, University of Rochester, Box 270168, Rochester, NY 14627 USA

**Keywords:** Breast cancer, Brain metastasis, Fluoxetine, Blood–brain barrier

## Abstract

**Background:**

Despite advances in the treatment of primary breast tumors, the outcome of metastatic breast cancer remains dismal. Brain metastases present a particularly difficult therapeutic target due to the “sanctuary” status of the brain, with resulting inability of most chemotherapeutic agents to effectively eliminate cancer cells in the brain parenchyma. A large number of breast cancer patients receive various neuroactive drugs to combat complications of systemic anti-tumor therapies and to treat concomitant diseases. One of the most prescribed groups of neuroactive medications is anti-depressants, in particular selective serotonin reuptake inhibitors (SSRIs). Since SSRIs have profound effects on the brain, it is possible that their use in breast cancer patients could affect the development of brain metastases. This would provide important insight into the mechanisms underlying brain metastasis. Surprisingly, this possibility has been poorly explored.

**Methods:**

We studied the effect of fluoxetine, an SSRI, on the development of brain metastatic breast cancer using MDA-MB-231BR cells in a mouse model.

**Results:**

The data demonstrate that fluoxetine treatment increases the number of brain metastases, an effect accompanied by elevated permeability of the blood–brain barrier, pro-inflammatory changes in the brain, and glial activation. This suggests a possible role of brain-resident immune cells and glia in promoting increased development of brain metastases.

**Conclusion:**

Our results offer experimental evidence that neuroactive substances may influence the pathogenesis of brain metastatic disease. This provides a starting point for further investigations into possible mechanisms of interaction between various neuroactive drugs, tumor cells, and the brain microenvironment, which may lead to the discovery of compounds that inhibit metastasis to the brain.

**Electronic supplementary material:**

The online version of this article (doi:10.1186/1471-2407-14-598) contains supplementary material, which is available to authorized users.

## Background

Despite recent advances in the treatment of primary breast cancer tumors, the incidence of fatal metastatic events remains high. Brain metastasis represents a particularly challenging complication of breast cancer. It is estimated that 10-15% of breast cancer patients have symptomatic brain metastases [1, 2] and as many as 30% of patients reveal brain metastases on autopsy [3, 4]. The brain provides a unique microenvironment for tumor growth. It is a particularly difficult therapeutic target due to the complexity of brain function as well as the reduced ability of therapeutic agents to cross the blood–brain barrier (BBB) [5]. In fact, many of the newest and most effective treatments for primary tumors are ineffective in treating breast tumor metastases in the brain [1, 5]. It is becoming increasingly clear that prevention and treatment of metastatic brain tumors requires a better understanding of the mechanisms that determine complex interactions between this unique metastatic milieu and tumor cells [2].

In this study we explore the mechanisms that underlie brain metastases by investigating possible effects of antidepressant drug treatment on their development. We present evidence that a selective serotonin reuptake inhibitor (SSRI), fluoxetine, facilitates increased brain-specific formation of breast cancer metastases in a mouse model of the disease. This is accompanied by increased permeability of the BBB and elevated production of pro-inflammatory cytokines, indicating that fluoxetine treatment may promote the entry of cancer cells into the brain via changes in the function of the BBB. This provides important insight into the mechanisms governing breast tumor metastasis to the brain, and possible ways to manipulate those mechanisms in order to reduce brain metastases. This approach has additional clinical relevance because it has been well documented that up to 25% of women with breast cancer suffer from clinical depression, a much higher percentage compared to the incidence observed in the general population [6, 7]. As a result, antidepressant drug use among breast cancer patients can be as high as 50% [8]. The SSRIs in particular have found widespread use in the clinical management of breast cancer-associated depression, hot flashes, and chemo brain [9, 10]. Recently, however, there has been increasing concern about pharmacologic interactions between several SSRI antidepressants and anti-tumor medications used in breast cancer therapy [11, 12]. Several studies indicate that simultaneous administration of these drugs may lead to decreased anti-tumor therapeutic effectiveness and increased risk of recurrent breast cancer or death, due to drug competition for binding sites at the relevant metabolic liver enzymes [13, 14]. Even though these reports warrant further experimental validation that considers genetic factors, patient drug compliance, and population dynamics [15, 16], there is no doubt that any clinical approach to the prevention and treatment of primary and metastatic breast cancer must take into account possible adverse effects of prescription drug use.

## Methods

### Cells

For intracardiac and tail-vein injections, we used the MDA-MB-231BR-GFP (231BR) human cell line that exhibits an ability to metastasize to the brain [17], a generous gift from Dr. P. Steeg. Cells were maintained in DMEM supplemented with 1% penicillin-streptomycin mixture. A YFP-expressing CNS-1 rat glioma cell line was used for intracranial injections, a generous gift from Dr. R. Mathews [18]. CNS-1 cells were grown in RPMI 1640 medium with 100 μg/ml of hygromycin B. All cell growth media were supplemented with 10% fetal bovine serum (FBS). Cells were regularly checked for mycoplasma contamination, with consistently negative test results.

### Fluoxetine administration and cell injection

All animal experimental protocols were approved by the University of Rochester Committee for Animal Research. Fluoxetine was added at 200 mg/L into drinking water supplied to adult female Nu/Nu mice (Charles River Laboratories) 21 days before either intracardiac or tail-vein injections, and continued during the 3-week survival period. For stereotactic injections into the brain parenchyma, animals were placed on dietary fluoxetine at 200 mg/L for 4 weeks before the cell injections; fluoxetine administration continued for 1 additional week, at which time brains were harvested. 231BR or CNS-1 cells were re-suspended in cold DPBS containing 0.5% FBS, and placed on ice prior to injection. *Intracardiac injections:* After anesthesia with Avertin, we injected 10^5^ 231BR cells into the left cardiac ventricle. Placement of the needle into the left ventricle was confirmed by the presence of pulsating arterial blood. *Tail vein injections:* Mice were placed into a mouse restrainer (Braintree Scientific) and injected with 10^6^ 231BR cells into a tail vein. At the end of each series of injections, cell viability was determined by Trypan Blue staining. Mice were weighed before and after experiments and checked for behavioral abnormalities every three days. No pathologic changes were detected in this study. *Intracranial injections:* Animals were anesthetized with isoflurane and placed into a stereotactic apparatus. A craniotomy was made, and 10^4^ CNS-1 cells were introduced into the frontal cortex of Nu/Nu adult female mice.

### Fluoxetine and norfluoxetine quantification by liquid chromatography mass spectrometry (LC-MS/MS)

Mice were treated with 200 mg/L of fluoxetine in drinking water for 30 days. 100 μl of serum was collected at day 0 and every 10 days throughout the fluoxetine treatment. SRMs for fluoxetine and norfluoxetine were performed by direct infusion in the positive mode using 50% methanol with 0.1% formic acid. The parent ion m/z, fragment ion m/z, collision energy, and tube lens voltage for the two compounds were 296.1 m/z. 134.1 m/z, 5, 68 for fluoxetine; and 310.1 m/z, 44.3 m/z, 13, 66 for norfluoxetine. To extract the compounds from serum, 5 volumes of acetonitrile (ACN) were added to the serum (500 μl of ACN to 100 μl of serum), followed by vortexing for 2 min and centrifugation at 16,000 g for 5 min at 4°C. The supernatant was collected and dried down in a SpeedVac. The dried material was reconstituted in 100 μl of 50% methanol, and 10 μl was injected for the LC-MS/MS run. LC-MS/MS runs was performed at 40°C on a Thermo Quantum Access Max triple quadropole mass spectrometer, with a Dionex Ultimate 3000 UPLC, configured with a 150 × 2.1 mm Accucore RP-MS column. The solvent system used 0.1% formic acid as solvent A and 100% methanol as solvent B, with a gradient elution run, beginning with 30% B for 0.5 minutes, ramping to 95% B over 1.5 minutes, holding at 95% B for 1 minute, and returning to 30% B in 0.25 minutes, with a final 30% B equilibration step for 2 minutes. Raw data files were imported into LCQUAN software, including a standard curve spanning concentrations of 10 nM - 3.16 μM, extracted from serum for fluoxetine and norfluoxetine. Area under the curve analysis was used to quantify the compounds in unknown samples.

Additional file 1: Figure S1A reveals that after 10 days of treatment, the mean concentration of fluoxetine reached 128 ng/ml, with the range of 55-243 ± 16 ng/ml. After 20 and 30 days of fluoxetine administration, the mean fluoxetine levels were 160 and 178 ng/ml, with the range of 80-306 ± 25 and 24-363 ± 39 ng/ml, respectively. The mean norfluoxetine concentration at the 10-day time point was 282 ng/ml, with the range of 140-479 ± 41 ng/ml, whereas at the 20 and 30 day interval, the mean norfluoxetine levels were 364 and 414 ng/ml, with the range of 74-532 ± 41 and 153-579 ± 46 ng/ml, respectively (Additional file 1: Figure S1B). The serum levels of fluoxetine were within the range reported previously for human serum samples [19]. However, norfluoxetine concentration reached ~ twofold higher levels than in human populations [19], probably due to the differences in metabolic transformation of the parent drug in mice versus humans.

### Immunohistochemistry and image analysis

To quantify brain metastasis, mice injected intracardially with 231BR cells were perfused with 4% paraformaldehyde. The brains were serially sectioned in the coronal plane at 50 μm. Sections were viewed on an AX70 Microscope (Olympus, Center Valley, PA) using an epifluorescence setup. Digital images were obtained using a MicroFire camera (Optronics, Muskogee, OK) and Image Pro software (Media Cybernetics, Bethesda, MD). Images were analyzed in ImageJ by a blinded observer. As reported previously in the literature [20], we classified visible metastases as “macrometastases” or “micrometastases” depending upon their size. Specifically, a cluster of cells that was greater than 100 μm in greatest extent was counted as a single “macrometastasis” while any cells in a cluster smaller than 100 μm in extent were defined as multiple “micrometastases” and counted individually. To quantify lung metastasis, lungs were perfused with 4% paraformaldehyde and embedded in paraffin. 5 μm serial sections were cut through the lungs at 300 μm intervals and stained with hematoxylin-eosin. The number of lung metastases was determined in 4–6 tissue sections per animal by a blinded investigator using an AX70 Microscope (Olympus, Center Valley, PA) in trans-illumination mode. To investigate brain-resident tumor growth, the brains of mice injected with CNS-1 tumors were serially sectioned at 50 μm. The sections were imaged by a blinded observer as described for 231BR cells above, and images were analyzed in ImageJ. Three measures were used to quantify CNS-1 tumor growth: the number of brain sections containing cells, the total number of tumor-containing pixels in the sections, and the maximum width that the cells spread perpendicular to the initial injection track. Imaging parameters and thresholds were kept constant between sections.

For immunohistochemistry (IHC), sections were washed in 0.1 M phosphate buffered saline (PBS), followed by incubation in 1% hydrogen peroxide to block endogenous peroxidase activity. Next, tissue was incubated in blocking solution containing 0.3% Triton-X and 5% normal donkey serum (NDS) in 0.1 M PBS. After an additional wash, the sections were incubated for 48 h in a humidified chamber at 4°C in primary antibody solution containing one of the following antibodies: rabbit anti-Iba-1 (1:500, Wako Pure Chemical Industries, Richmond, VA); mouse anti-IA/IE (1:200, BD Pharminogen, San Jose, CA); mouse anti-CD11b (1:200, AbD Serotec, Raleigh, NC); mouse anti-CD45 (1:300, AbD Serotec, Raleigh, NC); mouse anti-CD68 (1:800, Abcam, Cambridge, MA); rabbit anti-GFAP (1:1500, Abcam, Cambridge, MA); and Wisteria Floribunda Lectin (WFA) (1:500, Vector Laboratories). The sections were subsequently washed and incubated for 4 h at room temperature with either of the following secondary antibodies: Alexa Fluor 594 donkey anti-rabbit IgG (1:500) or Alexa Fluor 594 donkey anti-mouse IgG (1:500) (Molecular Probes, Carlsbad, CA). The sections were washed, mounted, and cover-slipped using ProLong Gold Antifade Reagent (Molecular Probes, Carlsbad, CA).

Sections were viewed on an AX70 Microscope (Olympus, Center Valley, PA) using an epifluorescence setup. Digital images were obtained using a MicroFire camera (Optronics, Muskogee, OK) and Image Pro software (Media Cybernetics, Bethesda, MD). Images were analyzed by a blinded observer using ImageJ. To determine the amount of glial staining in relation to distance from 231BR metastases, we created binary masks of tumors and glial staining. The tumor mask was then expanded iteratively by one pixel and the number of stained pixels within the region defined by the tumor mask was measured to produce the fraction of stained pixels as a function of distance from the edge of the tumor. All measurements were confined to the brain area in which the tumor resided to correct for differences in glial expression between brain areas. Tumors in control and fluoxetine groups were not statistically different in size for all stains. WFA antibody was used to visualize perineuronal nets in brain sections from animals that were injected with 231BR cells. To quantify WFA staining, background subtracted normalized average pixel intensity value was determined for various brain regions and compared between the control and fluoxetine groups.

### Thinned skull imaging

Chronic imaging of mouse visual cortex was performed using a thinned skull preparation as previously described [21], using GFP-M mice [22] that received 100 mg/L of fluoxetine in drinking water for 4 weeks. Briefly, a two-photon microscope with a Mai Tai laser (Spectra Physics) and a modified Olympus Fluoview 300 confocal unit was used. An Olympus LUMPlan fI/IR 20X/0.95NA was used to identify the binocular visual cortex based on cortical vasculature; an area containing brightly labeled neurons was chosen for imaging. 3D image stacks were obtained at high magnification to allow for dendritic spine reconstruction in layers 1 and 2 of the visual cortex. After the initial imaging session, the scalp was sutured and the animals were returned to the animal facility. The animals were re-anesthetized 4 days later and the same area was identified based on the blood vessel and dendritic patterns [21]. 3D image stacks of the same dendritic regions were again obtained at high magnification. The percentage of lost and new spines was determined relative to the total number of spines present in the initial imaging session using ImageJ.

### Proliferation and migration assay

For proliferation assays, 231BR cells were plated at 20,000 per well and incubated for 6 h to allow cells to adhere. The medium was replaced with DMEM containing fluoxetine at 1–5000 ng/ml. Cell numbers counted after 24, 48, and 72 h of incubation. Results are representative of two independent experiments. A migration assay was performed using the FluoroBlok 24-well insert system with 8.0 μm pore size (BD Biosciences, Bedford, MA). 231BR cells were grown for 48 h in DMEM containing various fluoxetine concentrations, trypsinized, counted, and seeded in serum-free DMEM/fluoxetine mixture onto the apical side of the insert at 50,000 per well. DMEM/fluoxetine with 10% FBS was added as a chemoattractant to the basal chamber. Following overnight incubation at 37°C in 5% CO_2_, cells were stained with calcein AM and then read on a bottom reading fluorescent plate reader.

### Evan’s Blue spectroscopy

Mice were injected via tail vein with 100 μl/10 g body weight of 2% Evan’s Blue in PBS. 1 hour after the injection, the animals were perfused with sterile isotonic saline, and the brains were removed and dried in a vacuum oven for 24 hours. Brain tissue was subsequently homogenized in a volume of PBS based on dry tissue weight, and then subjected to protein precipitation with trichloroacetic acid. The spectroscopic analysis of the supernatant was performed at 620 nm to determine Evan’s Blue absorbance.

### Quantitative RT-PCR

Animals were perfused with PBS containing 2 IU/ml of heparin. RNA was isolated from the brain tissue using TRIzol reagent, and 1 μg of the purified RNA product was subsequently reverse transcribed using Superscript III reverse transcriptase kit (Invitrogen). PCR was performed using TaqMan® Gene Expression Assays from Applied Biosystems, and the results were normalized to the expression of G3PDH.

### Cytokine immunoassay

Mice were perfused with PBS. Brain tissue was homogenized in RIPA buffer containing protease inhibitors (Thermo Scientific). 25 μl of protein extract was used in the subsequent immunoassay to determine cytokine expression. For the multiplex assay, a custom-made plate of mouse cytokines was used according to manufacturer’s instructions (EMD Millipore). Data were acquired on a FLEXMAP 3D system and analyzed with MILLIPLEX Analyst (EMD Millipore). Cytokine expression was determined in duplicate and subsequently normalized to sample protein concentration.

### Statistical analysis

Means and standard errors of the mean are presented, and significance was established using either Student’s *t*-test or analysis of variance (ANOVA). When ANOVA revealed statistical significance, multiple comparison *post hoc* analysis was performed to confirm differences between experimental groups. P < 0.05 was considered statistically significant.

## Results

### Fluoxetine increases the ability of breast cancer cells to metastasize to the brain

**Figure 1:**
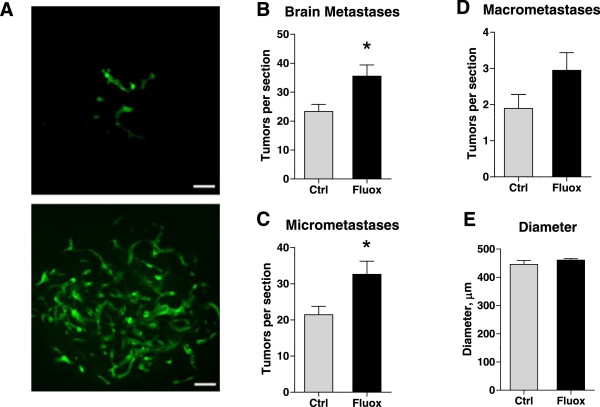
**Fluoxetine increases breast tumor metastasis to the brain.** Nu/Nu mice were treated with fluoxetine and injected with 231BR cells as described. **A)** Representative images of micrometastases (upper panel) and a macrometastasis (lower panel) in the brain of Nu/Nu mice 3 weeks after cell injection. Metastases were visualized in brain tissue by fluorescent microscopy. Note that the cells exhibited a tendency to localize perivascularly and form “sleeves” around blood vessels. Fluoxetine treatment increased the total number of metastases observed within the brain **(B)** as well as the number of brain micrometastases **(C)**, p < 0.05, *t*-test. **D)** While there was a trend towards an increase in the number of macrometastases, it did not reach statistical significance, p = 0.08, *t*-test. **E)** The diameter of macrometastases did not differ between the fluoxetine and control group. n = 11-12 per group. Scale bar: 50 μm.

To study the effects of fluoxetine on the ability of breast cancer cells to metastasize to the brain, we pretreated Nu/Nu mice with fluoxetine for three weeks prior to the intracardiac injection of 231BR breast cancer cells. Administration of fluoxetine in drinking water resulted in therapeutic concentrations in the serum as explain in the methods (Figure 1). Three weeks post-injection, metastases in fixed brain sections appeared either as isolated cells that could be readily distinguished and counted, which we term “micrometastases”, or as large groups of interconnected cells which could not be accurately distinguished and hence were counted as a single “macrometastasis” by our blinded observer (Figure 1A). Animals that received fluoxetine demonstrated a 52% increase in the total number of brain metastases compared with control: fluoxetine (n = 11), 35.54 ± 3.90 vs. control (n = 12), 23.33 ± 2.46 tumors/section, p = 0.02 (Figure 1B). This significant change in brain metastatic ability was largely due to increased incidence of micrometastases: fluoxetine, 32.59 ± 3.64 vs. control, 21.43 ± 3.64 tumors/section, p = 0.03, a 52% increase (Figure 1C). While not statistically significant, the same trend was evident for the number of macrometastases, with a 56% increase in the fluoxetine group: fluoxetine, 2.95 ± 0.49 vs. control, 1.89 ± 0.39 tumors/section, p = 0.08 (Figure 1D). The same outcomes have been observed in two independent experiments which have been pooled to produce the results described above.

**Figure 2:**
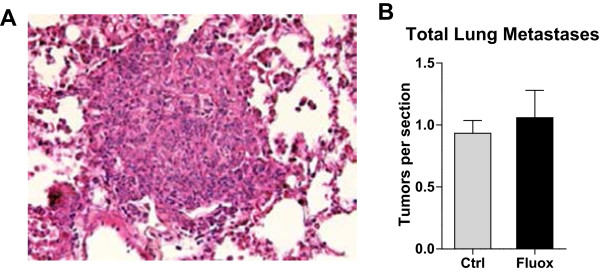
**Fluoxetine has no effect on breast tumor metastasis to the lungs.** Nu/Nu mice were treated with fluoxetine and injected with 231BR cells as described. **A)** Representative image of H&E staining of lung tissue containing metastasis 3 weeks after cell injection. **B)** Fluoxetine treatment did not affect lung metastasis development, p = 0.31, *t*-test. n = 5 per group.

Fluoxetine is a neuroactive substance suggesting that its effects may be brain-specific. In addition, 231BR cells have been selected for their preferential metastatic affinity to the brain. However, fluoxetine treatment may have altered metastatic targeting of 231BR cells and modified their potential to produce tumor growth elsewhere. To investigate this, we determined whether metastasis to another organ, the lung, was affected by fluoxetine treatment. Animals were treated as above and 231BR cells were then injected via the tail vein. Mouse lungs were removed after a 3 week survival period during which the animals continued to receive fluoxetine treatment. The tissue was fixed, paraffin embedded, serially sectioned, and stained with hematoxylin/eosin. The number of metastases in the lungs (Figure 2A) was determined using light microscopy. As shown in Figure 2B, fluoxetine treatment did not affect the ability of breast cancer cells to produce lung metastases, with 1.06 ± 0.22 vs. 0.93 ± 0.10 tumors/section in the fluoxetine and control groups, respectively, p = 0.31, suggesting that fluoxetine affects the entry of cells *specifically* into the brain rather than causing a non-specific increase in the cancer cells’ ability to survive within and/or extravasate from the vasculature.

### Proliferative and migration capacity of 231BR cells is not affected by fluoxetine

**Figure 3:**
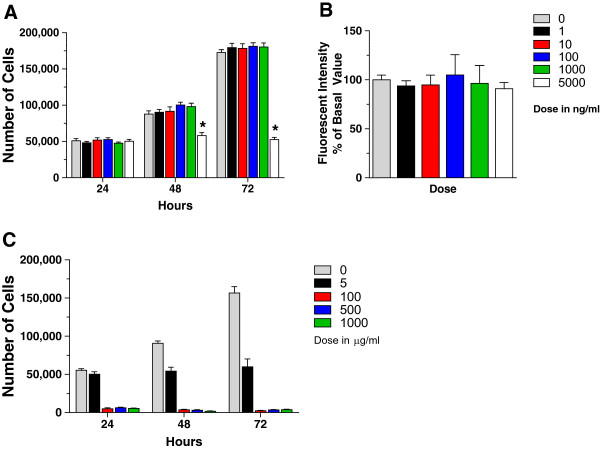
**The effect of fluoxetine on proliferation and migration of 231BR cells. A)** Fluoxetine treatment did not affect 231BR proliferation in vitro at 24, 48, and 72 h of incubation, except at 5000 ng/ml, when the drug caused cell growth arrest, p < 0.001, 2-way ANOVA with Bonferroni post-hoc analysis. n = 6-8. **B)** Migratory ability of 231BR cells in vitro was not affected by fluoxetine treatment, p = 0.98, one-way ANOVA, with Dunnett’s multiple comparison test. n = 8. **C)** At high doses, fluoxetine produces cytotoxic effect on 231BR breast cancer cells. Cell numbers were obtained after 24, 48, and 72 h of incubation with fluoxetine, as described. Whereas incubation with 5000 ng/ml (5 μg/ml) of fluoxetine caused an *arrest* in cellular proliferation starting at 48 hours, higher fluoxetine doses - 100 μg/ml, 500 μg/ml, and 1000 μg/ml - exhibited a clear toxic effect on 231BR cells. n = 4-7. Two independent experiments were conducted for each assay.

While the lack of a fluoxetine effect on lung metastasis suggests a brain-specific mechanism, we wanted to further rule out the possibility that fluoxetine interacts directly with 231BR cells to increase their proliferation and/or migration. Therefore, we performed *in vitro* proliferation assays in the presence of 1, 10, 100, 1000 or 5000 ng/ml of fluoxetine and measured 231BR proliferative activity at 24, 48, and 72 hours. Fluoxetine did not increase 231BR proliferation *in vitro* (Figure 3A). Incubation with 5000 ng/ml of fluoxetine caused an *arrest* in cellular proliferation starting at 48 hours (Figure 3A), with higher fluoxetine doses - 20 μg/ml, 100 μg/ml, 500 μg/ml, and 1000 μg/ml - exhibiting a clear toxic effect on 231BR cells (Figure 3C). Additionally, incubation with various concentrations of fluoxetine did not increase migration of 231BR cells *in vitro* (Figure 3B). These assays demonstrate that fluoxetine does not increase proliferation or migration of 231BR cells, thereby supporting our hypothesis that fluoxetine specifically affects the brain microenvironment.

### Fluoxetine treatment does not affect dendritic spine turnover and perineuronal nets

**Figure 4:**
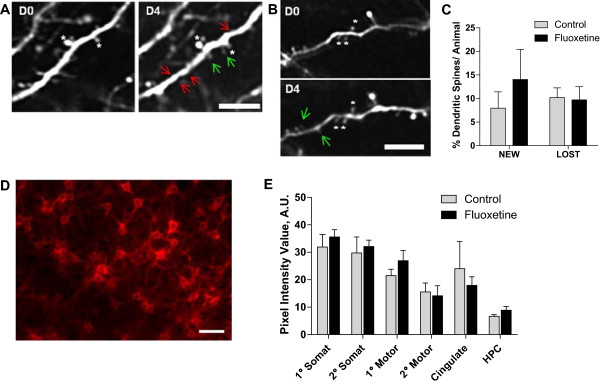
**The effects of fluoxetine treatment on dendritic spine turnover and PNNs. A-C**: Dendritic spine turnover was measured in adult mouse visual cortex in vivo, by imaging cortical dendrites four days apart using 2-photon microscopy. **A)** Representative images of dendritic spine turnover in control mice display both gain and loss of spines between imaging sessions (green arrows - new spines, red arrows - lost spines). **B)** Fluoxetine treated mice demonstrated similar numbers of new spines and lost spines. White asterisks denote reference spines between images. **C)** Quantification of dendritic spine turnover showed no significant difference between the percentage of new and lost spines in fluoxetine and control groups. Data are mean ± SEM, n = 4-5 per group. **D**-**E**: WFA antibody was used to visualize PNNs. **D)** PNNs are revealed around neuronal cell bodies in mouse cerebral cortex. **E)** Quantitative analysis of WFA staining in the control and fluoxetine groups was performed in primary (1°) and secondary (2°) somatosensory cortex (somat), 1° and 2° motor cortex (motor), cingulate cortex (cingulate), and the hippocampus (HPC). No significant changes were observed. n = 5-6 per group. Scale bar: **A**-**B**, 5 μm, **D**, 50 μm.

Our results suggest that fluoxetine acts on the brain microenvironment to enhance its capacity to foster metastasis. Two mechanisms that may contribute to this effect are: an enhanced growth of the established tumors within the brain parenchyma, or an increased ability for metastatic cells to penetrate the BBB. To examine the former possibility we examined the extracellular environment of the brain after fluoxetine treatment. Fluoxetine has been shown to modulate synaptic plasticity [23], a process that is dependent on remodeling of the brain extracellular matrix (ECM) [24]. ECM changes have the potential to influence breast tumor growth within the brain, since the invasion process is critically dependent upon the extracellular substrate [25]. To determine whether fluoxetine treatment altered the extracellular brain environment, we first assayed dendritic spine turnover *in vivo*, a process that is highly sensitive to brain ECM composition [26, 27]. GFP-M mice [22] were treated with fluoxetine for 4 weeks. Dendritic spines, which are the postsynaptic structures of the majority of excitatory synapses in the central nervous system, were imaged *in vivo* through a thinned-skull window on two separate imaging sessions spaced four days apart. As expected, examination of dendritic spine turnover revealed that animals in both the control (Figure 4A) and fluoxetine (Figure 4B) group demonstrate dynamic gain and loss of spines. However, quantitative analysis showed no significant difference in the percentages of either new or lost spines between the experimental groups (Figure 4C), suggesting that fluoxetine does not enhance structural plasticity at cortical synapses.

We also evaluated the direct effect of fluoxetine treatment on ECM composition, in particular on perineuronal nets (PNNs), a major component of the brain ECM that is rich in chondroitin sulfate proteoglycans [28]. PNNs have been implicated in modulating neuronal plasticity [29], and thus could be a prime target of fluoxetine action. Brain tissue of mice that received fluoxetine and were injected with 231BR cells was examined using a wisteria floribunda antibody (WFA) that recognizes PNNs (Figure 4D). The average fluorescent intensity of WFA staining was determined quantitatively across brain regions and compared between the control and fluoxetine groups. As shown in Figure 4E, WFA staining was highly variable throughout different brain regions, with primary and secondary somatosensory cortex exhibiting the highest level of PNN expression. Areas of primary and secondary motor cortex, as well as cingulate cortex, demonstrated somewhat lower WFA staining intensity, with hippocampus having the lowest expression of PNNs. However, a comparison within individual brain regions failed to reveal any difference between the control and fluoxetine experimental groups (Figure 4E). These results suggest that the increase in brain metastatic ability of breast cancer cells elicited by fluoxetine treatment is not modulated via large-scale changes in ECM either at synaptic sites or in PNNs.

### Effect of fluoxetine on tumor growth within the brain parenchyma

**Figure 5:**
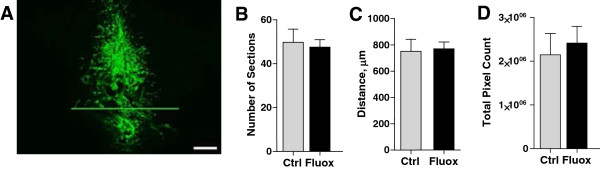
**Fluoxetine does not increase brain-resident tumor growth.** Nu/Nu mice treated with fluoxetine were injected with CNS-1 cells into the frontal cortex, as described. **A)** A representative image of a tumor formed after intracranial injection of CNS-1 cells. Scale bar: 200 μm. Three separate measures were used to quantify tumor growth. None of them showed significant effects of fluoxetine administration: number of sections containing CNS-1 tumors, p = 0.76 **(B)**; total pixel count analysis of brain sections with CNS-1 tumors, p = 0.68 **(C)**; tumor width, as determined by the average of four largest values from each animal, p = 0.86 **(D)**. n = 9-10 per group. Scale bar, 100 μm.

The lack of changes in dendritic spine dynamics and ECM structure suggests that fluoxetine may facilitate the entry of cancer cells into the brain rather than their subsequent growth within the brain parenchyma. This predicts that tumors growth is not altered by fluoxetine once cells are established within the brain. In support of this view, fluoxetine treatment did not affect the *size* of 231BR macrometastases: the average diameter was 1599 ± 17 a.u. in the fluoxetine group vs. 1547 ± 49 a.u. in the control group, p = 0.19 (Figure 1E). We hypothesized that if fluoxetine was changing the brain microenvironment to foster growth of established brain tumors, this should enhance the ability of any brain-resident tumors to grow within the brain. To test this, we performed stereotactic injections of a rat glioma cell line, CNS-1, into the frontal cortex of Nu/Nu mice, in order to examine whether fluoxetine would affect brain tumor development after introduction of malignant cells directly into the brain parenchyma. While intracranial injection of CNS-1 cells led to the development of brain tumors in mice (Figure 5A), 4 weeks of pre-surgical treatment with 200 mg/L of fluoxetine, followed by a 1 week survival period, did not affect brain tumor size when compared to the control group. Tumor spread, assayed by the number of sections containing CNS-1 cells, was comparable between the fluoxetine and control groups, 47.56 ± 3.24 and 49.8 ± 5.98, respectively, p = 0.76 (Figure 5B), as was the distance traveled by infiltrating tumor cells (771 ± 51 μm in the fluoxetine group vs. 751.4 ± 92 μm in the control group, p = 0.86, Figure 5C). The overall tumor size (total image pixel count per tumor), which may reflect the ability of tumor cells to proliferate within the brain, was comparable between treated and untreated groups, 2.148 ± 0.49 ×10^6^ vs. 2.148 ± 0.38 ×10^6^, respectively, p = 0.66 (Figure 5D). These findings suggest that fluoxetine may impact the ability of breast cancer cells to enter the brain, without altering their ability to infiltrate and spread once they have established metastatic foci within the brain parenchyma.

### Effect of fluoxetine on blood–brain barrier permeability

**Figure 6:**
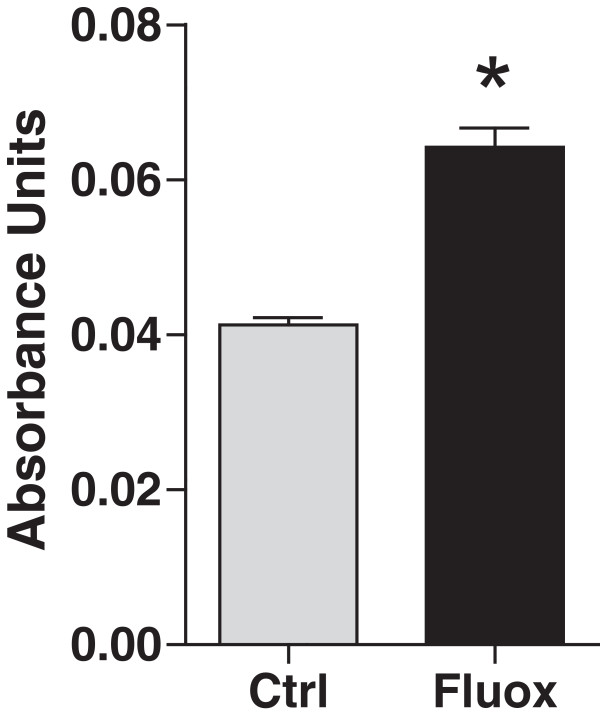
**Fluoxetine administration increases BBB permeability.** Fluoxetine was administered for 3 weeks before animals were injected intravenously with 2% Evan’s Blue solution. One hour after the injection, brain tissue was collected and processed as described. Tissue supernatants were analyzed by spectroscopy at 620 nm to determine Evan’s Blue absorbance. The results show significant effects of fluoxetine treatment. n = 6-7 per group, p < 0.0001.

A possible mechanism of increased brain metastatic breast cancer modulated by fluoxetine administration is a direct effect on BBB permeability. The BBB plays a critical role in the process of extravasation of cancer cells and determines their ability to seed the brain parenchyma [30, 31]. After a 3-week treatment with fluoxetine, we analyzed Evan’s Blue absorbance in brain extracts after tail vein injection of the dye to examine whether fluoxetine has any effect on BBB permeability. Brain extracts from animals that were treated with fluoxetine for 3 weeks demonstrate a statistically significant 54% increase in Evan’s Blue absorbance compared to the control group, p < 0.0001 (Figure 6). Thus, fluoxetine administration leads to changes in the BBB that promote increased permeability and may facilitate the increased entry of breast cancer cells into the brain.

### Fluoxetine stimulates production of pro-inflammatory cytokines

**Figure 7:**
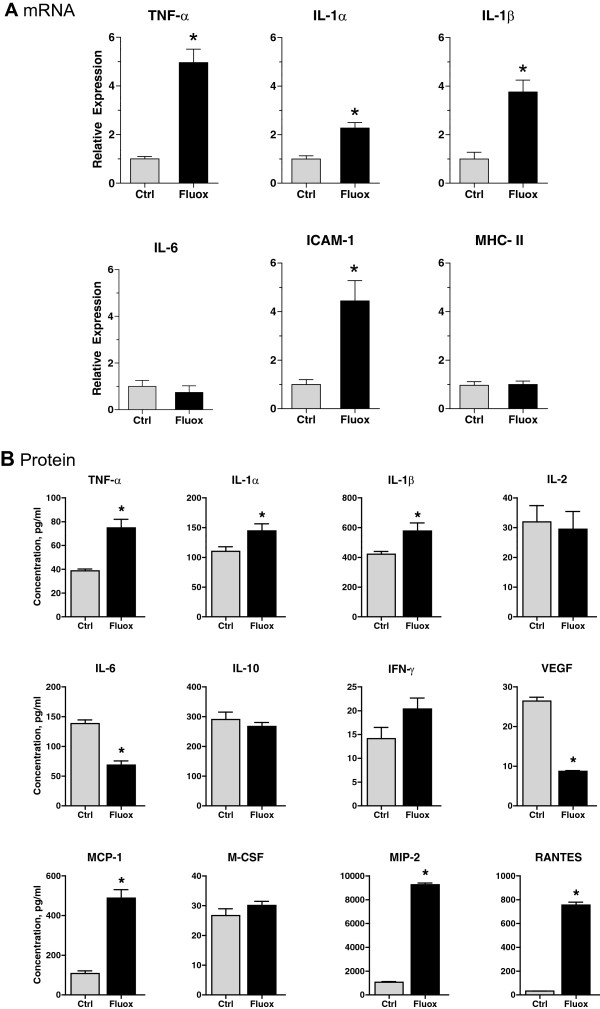
**Fluoxetine upregulates levels of pro-inflammatory cytokines. A)** To detect mRNA levels, brain tissue was collected after 3 weeks of fluoxetine treatment. mRNA was isolated, reverse transcribed, and subjected to real-time PCR analysis in order to determine expression levels of several pro-inflammatory markers. Experimental data were normalized to the expression of G3PDH, a housekeeping gene. n = 5 per group, p < 0.05. **B)** For protein analysis, after 3 weeks of fluoxetine administration, a custom-made mouse cytokine/chemokine panel was used to determine protein concentration of several pro-inflammatory markers in brain extracts from control and treated animals. Analyte expression was normalized to protein concentration in individual samples. n = 6 per group, p < 0.05.

A possible mechanism for changes in BBB permeability is production of cytokines that have been shown to modulate BBB function in models of injury, ischemia, and neurodegeneration [32, 33]. To determine whether fluoxetine treatment leads to increased expression of pro-inflammatory markers, mice were treated with fluoxetine, and brain extracts were analyzed using real-time PCR and multiplex ELISA. PCR analysis revealed that fluoxetine administration induced mRNA expression of several pro-inflammatory cytokines such as TNF-α, IL-1α, and IL-1β as well as an adhesion molecule ICAM-1, with levels increasing 4.96-, 2.27-, 3.76-, and 4.44-fold, respectively, p < 0.05 (Figure 7A). Transcription of two other pro-inflammatory molecules, IL-6 and MHC-II, was not significantly altered by fluoxetine treatment, p = 0.52 and 0.87, respectively. Protein analysis confirmed significantly elevated levels of TNF-α, IL-1α, and IL-1β, and demonstrated high levels of other cytokines - MCP-1, MIP-2, and RANTES, p < 0.05 (Figure 7B). The results of mRNA and protein expression assays demonstrate that fluoxetine can alter the inflammatory environment within the brain and stimulates cytokine production. This in turn may affect BBB permeability and lead to increased brain metastasis of circulating breast cancer cells.

### Fluoxetine enhances glial activation in the vicinity of brain metastatic tumors

**Figure 8:**
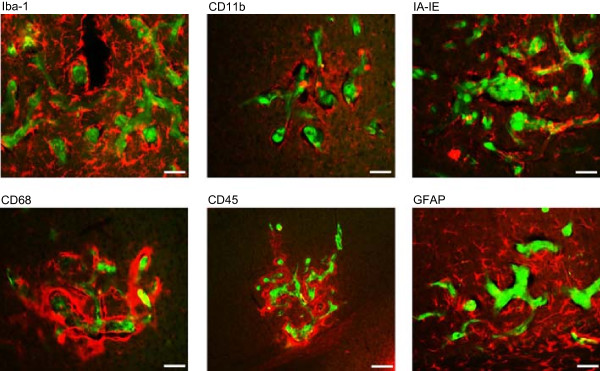
**Glial cells are activated in the vicinity of brain metastatic tumors.** Tissue sections from the control and fluoxetine groups were stained with antibodies against Iba-1, CD11b, IA-IE, CD68, CD45, and GFAP to determine the degree of microglial and astrocytic activation within 400 μm of metastatic breast cancer cells.

**Figure 9:**
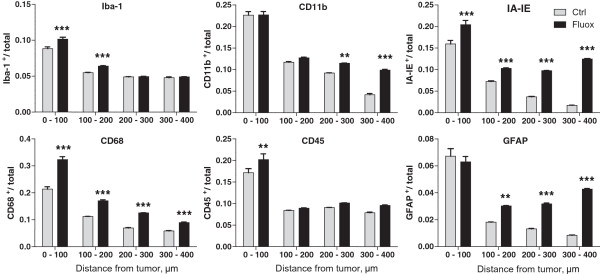
**Fluoxetine enhances glial activation in the vicinity of brain metastatic tumors.** Brain sections were stained with the following antibodies to assess glial activation - Iba-1, CD11b, IA-IE, CD68, CD45, and GFAP. Images of tumors were analyzed to compare the expression of these markers (fraction of total pixels that were immunopositive) within 400 μm of the edge of metastatic tumors, following fluoxetine treatment. *p < 0.05; **p < 0.01; ***p < 0.001, 2-way ANOVA with Bonferroni post hoc analysis. n = 20-24 tumors per group, 5–6 animals per group.

**Figure 10:**
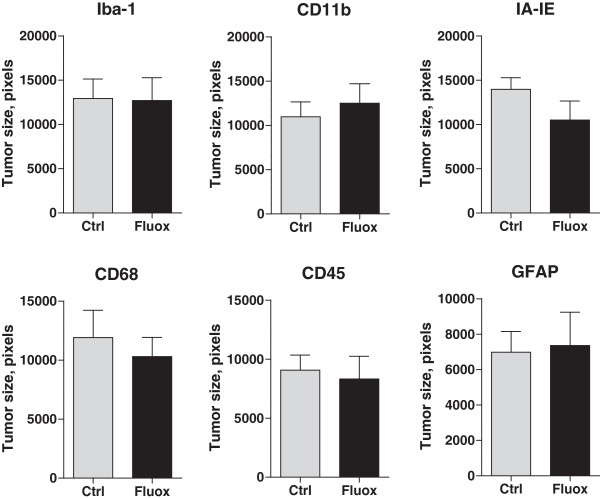
**Tumor size in samples analyzed for glial activation in the vicinity of brain metastases.** Tumor size was determined for each image used in the quantitative analysis of microglial and astrocytic activation. The comparison revealed no significant differences between the size of tumors between the control and fluoxetine groups. Iba-1, p = 0.95; CD11b, p = 0.58; IA-IE, p = 0.16; CD68, p = 0.59; CD45, p = 0.74; GFAP, p = 0.86, *t*-test. n = 20-24.

Microglia and astrocytes are two possible sources of pro-inflammatory markers that may affect the functioning of the BBB and thereby facilitate enhanced entry of tumor cells to the brain. To determine whether fluoxetine altered the activation pattern of glia around tumors, we stained brain sections with a number of antibodies specific for microglia and astrocytes (Figure 8). Both microglial and astrocytic markers were markedly elevated in proximity to the tumor in control animals, indicating an inflammatory response around metastases. Interestingly, fluoxetine treatment elevated the expression of both microglial and astrocytic markers showing that fluoxetine altered inflammatory signaling in response to metastasis (Figure 9). Signal intensity for microglial markers IA-IE and CD68 was significantly higher in the fluoxetine group throughout the entire area we examined (up to 400 μm distance from the tumor, p < 0.001). Other microglia-specific antibodies, Iba-1 and CD45, exhibited higher expression levels closer to the tumor, following fluoxetine administration (p < 0.001 and p < 0.01), whereas CD11b levels were higher between 200 and 400 μm away from the tumor (p < 0.01). In addition, staining intensity for GFAP, an astrocytic marker, was significantly higher between 100–400 μm in the fluoxetine treated animals compared to control, p < 0.01 (Figure 9). In each case the tumors examined were not significantly different in size in control and fluoxetine groups (Figure 10).

## Discussion

In this study we describe fluoxetine’s ability to increase the number, but not the size, of metastases in a murine model of breast tumor metastasis to the brain. This increase is accompanied by changes in the BBB and the inflammatory environment of the brain, with no detectable changes in the properties of the brain ECM. These results provide several insights into the possible mechanisms by which fluoxetine alters brain metastasis, and hence possible avenues for future therapeutic manipulation of the metastatic outcome.

### Fluoxetine and the brain ECM

Fluoxetine is thought to exert its anti-depressant effects by promoting brain plasticity, synaptogenesis and neurogenesis [23, 34]. These processes are critically dependent on the brain ECM, as is tumor invasion [35], suggesting that fluoxetine could achieve its metastasis-altering effects in part by remodeling the extracellular milieu of the brain [36]. We examined this possibility by focusing on an ECM component, the PNN, which has been shown to play a critical role in modulating plastic changes in the brain. PNNs are established during brain development, as inhibitory and excitatory circuits mature and the brain becomes less plastic [37]. Both enzymatic removal of PNNs and fluoxetine treatment enhance plasticity in the adult [23, 38]. However, we detected no significant change in PNNs in different brain areas after fluoxetine treatment, which suggests that the effects of fluoxetine on brain plasticity and metastasis are mediated through a different pathway. In agreement with this, synapse remodeling, a process that is highly sensitive to the extracellular environment [24, 27], was also not affected by fluoxetine. Given the apparent lack of fluoxetine-induced ECM remodeling in our brain metastasis model, it is not surprising that neither the size of breast tumor metastases established in the brain nor the size of resident glioma tumors appeared sensitive to fluoxetine treatment in our experiments. These results suggest that the effects of fluoxetine on the establishment of brain metastasis are not mediated via the brain ECM.

### Fluoxetine treatment and glia

The pathogenesis of the vast majority of CNS diseases, including depression, is mediated, at least in part, by inflammatory processes. Although fluoxetine acts as a serotonin reuptake inhibitor, it also has strong effects on peripheral immune cells [39] and brain resident immune cells [40–42]. We observed fluoxetine-induced alterations in expression of several cytokines indicative of glial activation, and observed that fluoxetine enhanced glial activation in the vicinity of established brain metastases. This suggests that fluoxetine can alter inflammatory signaling in vivo, and that this alteration may be a mechanism by which fluoxetine elevates breast tumor metastasis to the brain in our murine model.

The contribution of microglia and astrocytes to the pathology of brain resident tumors has been well documented [43–45]. However, their involvement in metastatic events is less clear. Several studies have described activated glia associated with metastastic lesions in the brain parenchyma, suggesting an important role for these cells in metastatic growth within the brain [25, 43]. While the immune function of these cells could contribute to defending the brain against cancer cell invasion, it is becoming clear that brain tumor cells can co-opt glia to promote tumor growth and invasion. Tumor cells and glia undergo a complex molecular cross talk that influences glial behavior and subsequent tumor progression [44, 45]. Activated glia can produce multiple cytokines, chemokines, and enzymes that lead to increased tumor invasion, including IL-1β and TNF-α [46], markers that were upregulated in this study. Surprisingly, the elevated levels of pro-inflammatory markers after fluoxetine treatment did not affect the growth of gliomas or breast tumor metastasis already established in the brain, despite the fact that many of these inflammatory molecules have been shown to play a significant role in tumor survival and angiogenesis [47, 48]. Possibly, the source or extent of fluoxetine-driven expression of these molecules is such as to change the entry of cells into the brain but not affect their subsequent growth.

It is interesting to note that in our study, chronic fluoxetine treatment *increased* inflammatory marker expression in the brain, while other studies examining peripheral and brain effects of fluoxetine have reported anti-inflammatory effects with *reduced* expression of markers such as TNF-α [40–42], although inflammatory effects of fluoxetine were also observed [41]. There are several possible reasons for this discrepancy. First, the majority of studies focus on the effects of fluoxetine within the context of pathological inflammation due to either LPS injection [40] or CNS disease [42]. Thus the effects on baseline inflammatory state have not been examined. Second, most studies examining inflammatory markers have focused on *in vitro* settings where cells behave differently than they do *in vivo*[41]. Our data suggest that fluoxetine, at neuroactive doses, can increase inflammatory signaling *in vivo* in the *absence of pathological changes in the brain* and this in turn may affect breast tumor metastasis.

### Inflammation and BBB

Glial cells, and astrocytes in particular, are critical elements of the BBB and could influence tumor cell entry into the brain through its manipulation. Glia-derived cytokines and proteases have been implicated in promoting cancer cell navigation through the BBB [32, 33, 49]. Interestingly, brain-resident glia are frequently localized to the sites of cancer cell arrest in brain capillaries [25]. The intimate relationship between glia and tumor cells that have not yet entered the brain might imply a role for glia in shepherding tumor cells through the BBB. In addition, glial cells also produce MCP-1, MIP-2, and RANTES (all of which were increased after fluoxetine treatment) that could promote metastasis indirectly by stimulating infiltration into the brain of peripheral cells with pro-tumor activities such as myeloid-derived suppressor cells (MDSC), tumor-associated macrophages (TAM), and tumor-associated neutrophils (TAN). These cells may in turn contribute to the vicious circle of the pro-invasion phenotype created by fluoxetine administration, via additional secretion of IL-1β, TNF-α, and other cytokines. Moreover, in the process of infiltrating the brain parenchyma, MDSCs, TAMs, and TANs may create a “back door” whereby cancer cells in the immediate vicinity can accompany the infiltrating cells as they leave the capillaries.

### Fluoxetine treatment and the BBB

A surprising result of fluoxetine administration is significantly increased BBB permeability even in the absence of circulating tumor cells. These data suggest that fluoxetine may facilitate the entry of breast cancer cells into the brain by affecting the function of the BBB directly rather than enhancing the transport of tumor cells specifically. In other disease models such as CNS trauma, ischemia and neurodegeneration, a number of pro-inflammatory mediators are released by brain parenchymal cells, including endothelial cells and glia [50]. These mediators, including IL-1β and TNF-α, increase BBB permeability [32, 49] via altered expression of tight-junction proteins as well as increased production of reactive oxygen species and metalloproteases [51]. Therefore, the increased expression of IL-1β and TNF-α that we observed after fluoxetine administration may directly lead to the impairment of BBB function and increased permeability of the barrier, thus precipitating increased brain metastasis.

Another important step in tumor cell extravasation is cell arrest within the blood vessels of the brain. Paracrine stimulation by pro-inflammatory molecules such as TNF-α, IL-1β, and MIP-2, leads to increased synthesis of chemokines and expression of cell adhesion molecules such as ICAM-1, E-selectin, and vascular cell adhesion molecule-1 (VCAM-1) by cerebrovascular endothelial cells [52], which may increase anchorage of tumor cells and eventually lead to facilitated cellular invasion from the circulation into the brain [53]. These same changes may directly or indirectly lead to increased ability of MDSCs, TAMs, and TANs to enter the brain and further influence tumor cell entry across the BBB. Additionally, inflammatory expression may influence the survival of tumor cells within the vasculature and thus enhance the probability of brain metastasis.

## Conclusions

Our data provide the first experimental evidence that a neuroactive drug can promote increased entry of cancer cells into the brain parenchyma. The results of this study suggest a novel drug-induced, brain-specific mechanism whereby permeability of the BBB is altered by 1) the effect of fluoxetine on cellular components of the brain microenvironment to stimulate production of pro-inflammatory cytokines that can in turn modulate BBB function, 2) direct effect of fluoxetine on the components of the barrier, or 3) a combination of these two mechanisms. These findings suggest that neuroactive drugs used to treat depression and chemo brain in patients need to be carefully screened for unexpected effects on brain metastasis. In addition, they open new opportunities in the search for pharmacologic drugs that would inhibit brain metastasis by restricting permeability of the BBB or, conversely, would improve the delivery of therapeutic agents to the brain by opening up the BBB. Such drugs would have the advantage of targeting the brain rather than the heterogeneous and rapidly mutating tumor cell and could be used to limit brain-specific metastasis of many different primary tumor types.

## Electronic supplementary material

Additional file 1: Figure S1: Fluoxetine reaches therapeutically relevant levels in mouse serum. Nu/Nu mice were treated with fluoxetine for 30 days as described. Mouse serum was collected at day 0 and every 10 days throughout the experiment. The concentration of fluoxetine and its major metabolite, norfluoxetine, was determined by LC-MS/MS. A) The mean fluoxetine concentration reaches 128 ng/ml after 10 days of treatment and remains at therapeutic levels at 30 days. B) The mean norfluoxetine level after 10 days is 282 ng/ml, and continues to increase. (PDF 15 KB)
