# Supplementary figures and images for: Fluoxetine modulates breast cancer metastasis to the brain in a murine model
Source: BMC Cancer. 2014 Aug 16;14:598. doi: 10.1186/1471-2407-14-598 (PMC4242485; doi:10.1186/1471-2407-14-598)

Supplementary Figure 1.

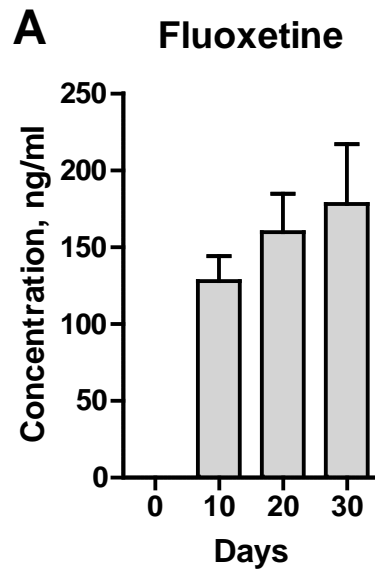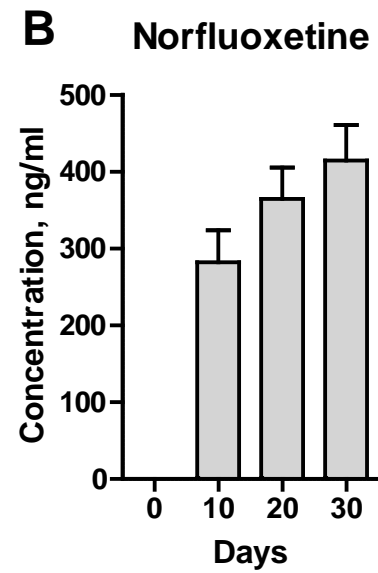

Supplement: Supplementary file 1 — Additional file 1: Figure S1: Fluoxetine reaches therapeutically relevant levels in mouse serum. Nu/Nu mice were treated with fluoxetine for 30 days as described. Mouse serum was collected at day 0 and every 10 days throughout the experiment. The concentration of fluoxetine and its major metabolite, norfluoxetine, was determined by LC-MS/MS. A) The mean fluoxetine concentration reaches 128 ng/ml after 10 days of treatment and remains at therapeutic levels at 30 days. B) The mean norfluoxetine level after 10 days is 282 ng/ml, and continues to increase. (PDF 15 KB) [file 12885_2014_5016_MOESM1_ESM.pdf]
